# Unilateral non-rotated left kidney with vascular and ureter variations in a cadaver: a unique case report with embryological and educational aspects

**DOI:** 10.1590/1677-5449.200156

**Published:** 2021-09-15

**Authors:** Kusum Gandhi, Judith Jaison, Eti Mantri

**Affiliations:** 1 All India Institute of Medical Science – AIIMS, Department of Anatomy, Bhopal, Madhya Pradesh, India.

**Keywords:** kidney, non-rotation, ureter, renal arteries, partial nephrectomy, rim, sem rotação, ureter, artérias renais, nefrectomia parcial

## Abstract

The kidneys and ureters are retroperitoneal structures in the upper part of the paravertebral gutters, tilted against the structures on the sides of the lowest two thoracic and upper three lumbar vertebrae, so that their anterior and posterior surfaces face antero-laterally and postero-medially, respectively. Congenital anomalies of the urinary tract are often the underlying cause of renal pathologies; 40% of these pathological conditions are due to variations in location, shape, and size of the kidney(s), calyces, ureter, or bladder. This case report describes the presence of a unilateral non-rotated left kidney with vascular and ureter variations found during routine cadaveric dissection for medical graduates. Alterations in rotation of the kidney and its relation to structures at the hilum have great clinical significance when conducting surgical procedures like partial nephrectomy, nephron sparing surgery, and renal transplantation.

## Resumo

Os rins e o ureter são estruturas retroperitoneais localizadas na parte superior do sulco paravertebral, inclinados contra as estruturas laterais das duas vértebras torácicas inferiores e das três vértebras lombares superiores. Assim, as superfícies anteriores e posteriores estão nas porções anterolateral e posteromedial, respectivamente. As anomalias congênitas do trato urinário frequentemente são causas subjacentes de patologias renais, e 40% delas ocorrem devido a variações no local, formato e tamanho dos rins, cálices, ureter ou bexiga. Este relato de caso descreve a presença de rim esquerdo unilateral sem rotação com variações vasculares e de ureter encontradas durante uma dissecção de cadáver rotineira com graduandos de Medicina. As alterações na rotação do rim e a sua relação com as estruturas no hilo têm grande importância clínica durante procedimentos cirúrgicos como a nefrectomia parcial, a cirurgia poupadora de néfrons e o transplante renal.

## INTRODUCTION

The kidneys and ureters are retroperitoneal structures in the upper part of the paravertebral gutters, tilted against the structures on the sides of the lowest two thoracic and upper three lumbar vertebrae, so that anterior and posterior surfaces face antero-laterally and postero-medially, respectively.^1^ Congenital anomalies of the urinary tract are often the underlying cause of renal pathologies; 40% of these pathological conditions are due to variations in location, shape, and size of the kidney(s), calyces, ureter, or bladder.^2^ Rotational anomalies are a rare entity that is not cited in most standard embryology textbooks and has important clinical implications from the surgical point of view, such as in percutaneous nephrectomy and in preoperative diagnostic evaluation of kidney donors.^3^

Kidney malrotation was found in only 1 of 939 autopsies and in 1 case per 390 hospital admissions.^1^^-^ 6 Renal malrotation is very commonly associated with an ectopic or fused kidney, but it may also occur in kidneys with complete ascent. Renal malrotation consists of non-rotation, incomplete rotation, reverse rotation (the hilum faces laterally), and hyper-rotation (the renal hilum faces ventrally, laterally or dorsally).^4^ Renal malrotation can be unilateral or bilateral, although the latter may be misdiagnosed as a horseshoe kidney.^1^^,^ 3 ^,^ 4 ^,^ 6 ^-^ 10

The primitive embryonic kidneys develop in the pelvis with the hila directed ventrally.^1^^,^ 6 Ascent of kidneys precedes the descent of developing gonads into the pelvis during 6^th^ to 9^th^ week of embryology period. To arrive at their definitive position, the kidneys undergo gradual ascension in the renal fossa along the dorsal aorta.^5^^-^ 10 During the process of ascent, the kidneys rotate 90 degrees around their longitudinal axis, so that the hilum faces medially.^1^^-^ 5

Kidney malrotation along with vascular and ureteral variation is an entity very rarely described in literature.^1^^,^ 6 Anatomical variations in the arterial, venous, and ureteral patterning of the kidneys are common; concomitant involvement of all these anatomical variants in a single individual appears to be much rarer. Arterial variants that diverge from the generalized pattern of paired renal arteries emerging laterally from the aorta, inferior to the level of the superior mesenteric artery, have been observed in approximately 30% of individuals.^11^^,^ 12 In the venous system, variations in the drainage pattern that diverge from the classically described paired vessels flowing into the inferior vena cava (IVC) have been well documented and can have an incidence rate similar to that of the arterial system.^13^ However, variation in the ureteral pattern is not as common as that found in the vascular system. Reports indicate that genetic factors contribute to ureteric variations^14^^,^ 15 which occur with an approximate incidence of 1%.^16^^,^ 17

The case study presented here describes concomitant variation in the rotation of the left kidney, its arterial supply, venous drainage, and ureter formation. Collectively, this case provides examples of important anatomic considerations that relate foundational anatomical science to educational practice. Ectopic location and unusual vasculature can predispose to iatrogenic trauma during interventional radiological and interventional laparoscopic procedures and emergency operations.^17^^,^ 18 Therefore, knowledge of the possibility of this anatomical variation will be of great help to clinicians and nephrologists in making a correct diagnosis and preventing complications during nephron sparing surgery and renal transplantation.

## CASE REPORT

Routine undergraduate dissection for medical students in our department revealed a unilateral non-rotated kidney on the left side in a 97-year-old male cadaver. The body was donated to our department for teaching and research purposes. The cause of death was cardiopulmonary arrest.

The right kidney was found to be normal in its location, shape, size, and ureter formation. Varied vasculature was observed in the form of branches of the renal artery and formation of the renal vein. The left kidney was found to be at a lower position than usual, at the level of the second, third, and fourth lumbar vertebrae. The inferior pole was at the level of the L4-L5 intervertebral disc. The kidney was broader near its middle third and inferior pole. The cratered hilum of the left kidney, instead of being at its medial border, occupied a large part of the anterior surface and was situated nearer to the lateral than the medial border. The renal vein and ureter were the two structures traversing through the hilum, but the renal artery was entering the renal parenchyma through the medial border.

**Figure 1:**
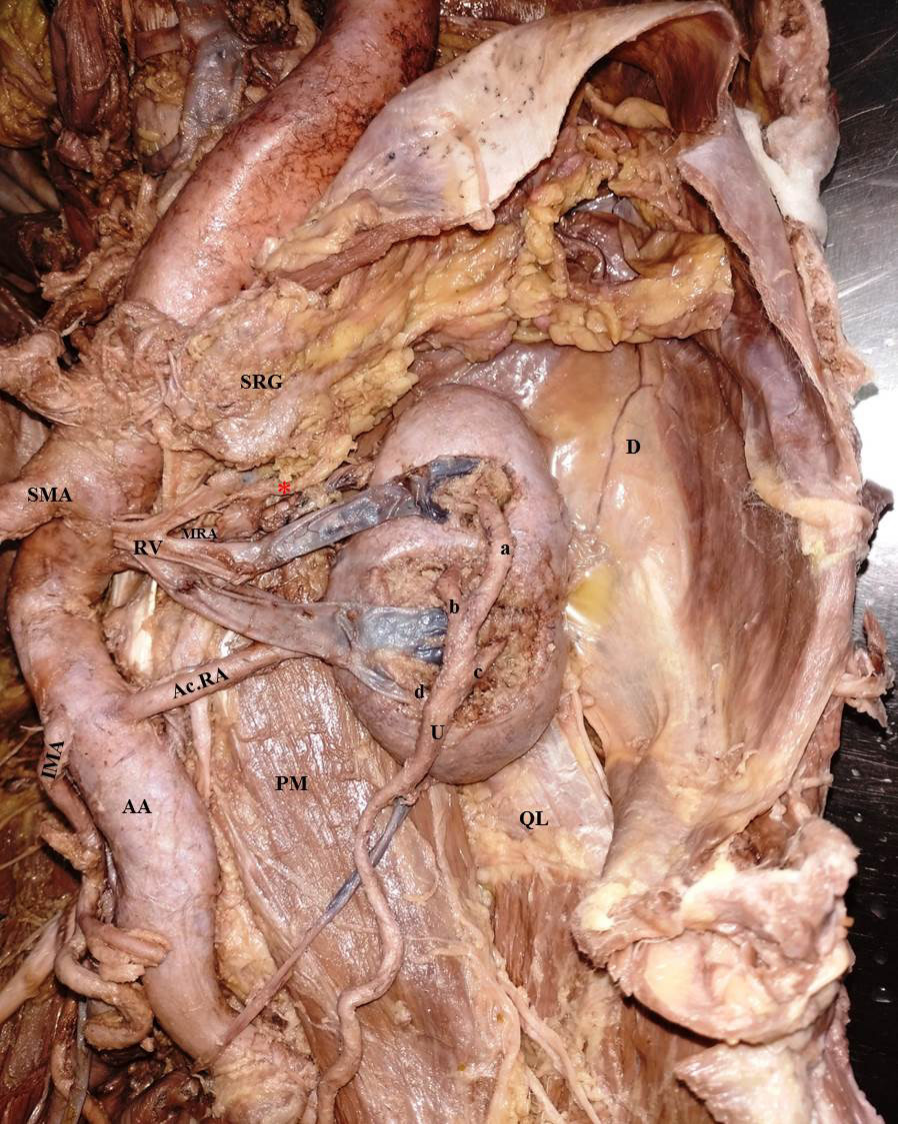
Shows left kidney with ventrally located hilum and the tubular ureter emerging through the cratered hilum. The two tributaries of the renal vein were medial to the ureter and anterior to the main renal artery. The main renal artery took origin from the tortuous aorta at the same level as the origin of the superior mesenteric artery. An additional accessory renal artery took origin from the abdominal aorta inferior to the inferior mesenteric artery. A well-defined renal pelvis was absent and the tubular ureter was formed of four tubular units, marked a, b, c, and d; (AcRA) Accessory Renal Artery; (MRA) Main Renal Artery; (RV) Renal Vein; (SMA) Superior Mesenteric Artery; (IMA) Inferior Mesenteric Artery; (PM) Psoas Major Muscle; (QL) Quadratus Lumborum; (U) Ureter; (D) Diaphragm.

The structures emerging from the hilum of the kidney mainly comprised the renal vein and the tubular ureter. The renal vein had two major tributaries, superior and inferior. The inferior tributary was formed by joining two tributaries emerging from the inferior region of the renal parenchyma and proceeded obliquely upward and medially to join with the superior renal vein. The left gonadal vein drained into this inferior tributary. The main trunk of the renal vein traversed its usual course between the abdominal aorta and superior mesenteric artery to drain into the inferior vena cava. There was no well-defined renal pelvis; instead the tubular ureter had four divisions entering into the renal parenchyma at the hilum of the kidney (labeled a, b, c, and d in Figure 1). The course of the ureter was then downward and medial to the urinary bladder.

**Figure 2:**
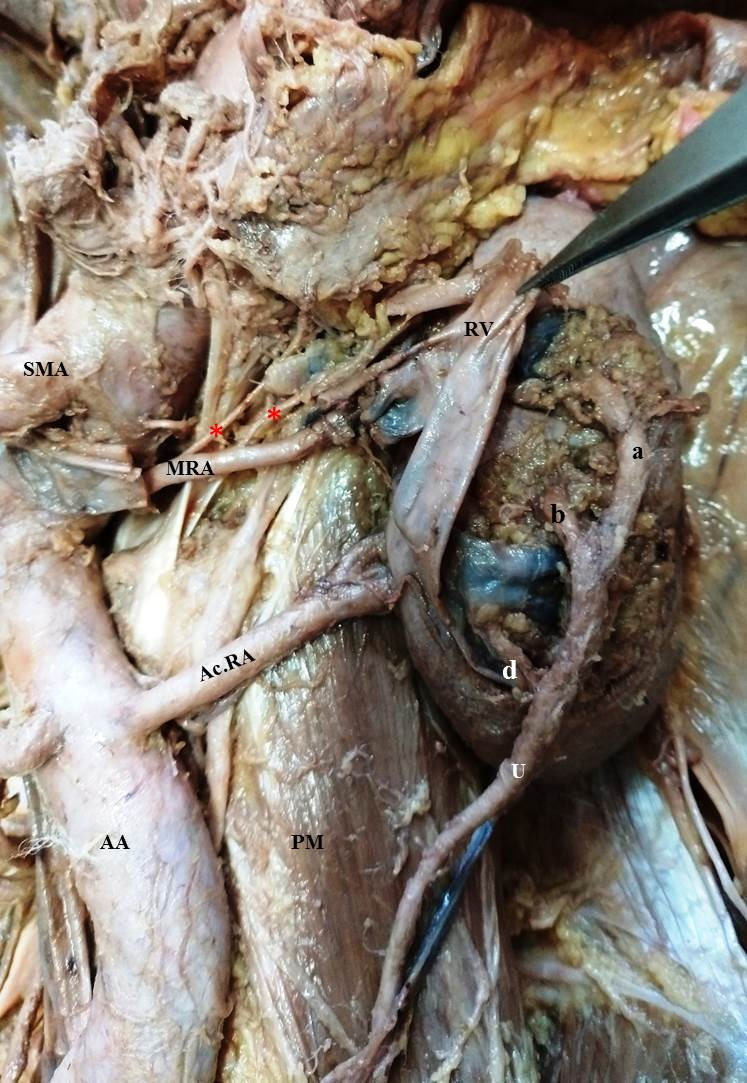
The left renal vein traversing anterior to the main renal artery has been reflected to expose the main renal artery and its branches to the left suprarenal gland (shown with red asterisks). Note that after providing branches to the suprarenal gland, the AcRA pierced the upper half of the medial border of the kidney. The AcRA was thicker in comparison to the MRA. (AA) Abdominal aorta; (AcRA) Accessory Renal Artery; (MRA) Main Renal Artery; (RV) Renal Vein; (SMA) Superior Mesenteric Artery; (IMA) Inferior Mesenteric Artery; (PM) Psoas Major Muscle; (QL) Quadratus Lumborum; (U) Ureter; (D) Diaphragm.

The abdominal aorta was tortuous all along its course in the abdomen. The Main Renal Artery (MRA) is described as a single vessel, at a more or less constant position opposite the renal hilus, from the abdominal aorta, and which continues undivided (except for several small branches- the inferior suprarenal, the perirenal and the ureteral arteries) in its straight course to the renal hilus. The MRA was thin and originated from the abdominal aorta at a level just inferior to the origin of the superior mesenteric artery, entering the kidney through its medial border after giving off 3 branches (represented by red asterisks in Figures 1 and 2) to the left suprarenal gland. The accessory renal artery (AcRA) arose from the aorta just lateral and inferior to the origin of the inferior mesenteric artery, entering the renal parenchyma through the medial border of the kidney. The AcRA divided into two segmental arteries before piercing the lower part of the medial border of the kidney (Figure 2). 

The case was observed in a body donated to our department for educational and research purposes. Body is accepted only after taking consent of the deceased/next of kin which is in accordance to Anatomy act of India.

## DISCUSSION

Congenital abnormalities of the kidney and urinary tract occur in approximately 3.3–11.1% of the population and account for about 50% of all congenital abnormalities.^19^ The most common renal malrotation is non-rotation, whereas hyper-rotation with the hilum facing laterally is the least common.^17^ Renal malrotation is usually asymptomatic and detected accidentally,^6^^-^ 8 although it may occasionally lead to ureteral distortion, urinary tract obstruction, urolithiasis, or urinary tract infection.^8^^,^ 10 Interestingly, renal malrotation is also accompanied by acute pyelonephritis and renal infarction in cases reported by Tsai et al.^20^

### Anatomical variations

Arterial variants have been observed in approximately 30% of individuals, although population-specific incidences have been shown to vary considerably.^11^^,^ 21 Studies documenting the major renal branches (rather than the branching pattern into the kidney parenchyma) suggest that arterial origins are possible along the entire abdominal aortic axis. Branches at or superior to the level of the superior mesenteric artery^21^ have been well documented. However, renal arteries entering the renal parenchyma through the medial border in a malrotated kidney are not well documented in the literature. As with the arterial system, variations in renal vein patterning are not uncommon. Studies with large sample sizes (e.g., >100) suggest that variations in venous patterning have an incidence of 23.5-30%, as observed through computed tomography (CT).^13^ Variations in ureteral patterning are not as common as those found in the vascular system, but reports indicate an approximate incidence of 1%.^16^^,^ 17 Interestingly, to the best of our knowledge, the pattern of ureter formation that we encountered in this case is not reported in the literature.

### Educational importance

Undergraduate and postgraduate students, who participate in dissection courses during their training, learn the basic features of human anatomy as well as anatomical variations. Distinguishing which variations have clinical significance requires both breadth and depth of knowledge, particularly within each clinical speciality.^22^ This case report will provide knowledge about unique anatomic variations of concomitant involvement of kidney, arteries, and ureter in the same individual. Recognizing these variations when planning appropriate investigation and treatment procedures enables students as well as clinicians to perform safe and uncomplicated surgical procedures.

### Embryological aspect

The anatomic variations of the current case collectively reflect important concepts related to the morphological development of the kidney, its collecting system, arterial supply, and venous drainage. During human development, three slightly overlapping renal systems are formed in a cranial to caudal fashion: pronephros, mesonephros, and metanephros.^23^ The pronephros is a temporary organ that regresses at the end of the fourth week of fetal life, while the mesonephros contributes to Bowman’s capsule and the Wolffian duct in adult kidneys. The metanephros appears in the fifth week of fetal life and will give rise to the adult kidney. The renal excretory units originate from the metanephric mesoderm and the collecting ducts from the ureteric bud that penetrates the mesodermal tissue.

The primordial kidneys are initially located within the pelvis with their hila directed anteriorly. The kidneys receive their arterial supply from the pelvic branch of the abdominal aorta and this orientation changes during development. Following growth and change in body length at the lumbar and sacral regions, the kidneys undergo a relative ascent in the abdomen as they shift to a more cranial position.^24^^,^ 25 This ascent is accompanied by a transitioning arterial supply originating from aortic branches at sequentially higher levels and medial rotation of the developing kidney. The lower vessels usually degenerate, but occasionally some remain, leading to anatomic variations in the adult. Unlike the arterial system, the venous system for the kidney arises from anastomotic connections between the developing cardinal veins.^25^ Briefly, at 5-6 weeks of development, the posterior and subcardinal veins exhibit anastomoses in a segment-like fashion that drain aspects of the early mesonephros. The subcardinal veins anastomose and coalesce at the midline, contributing to the prerenal portion of the IVC and what is generally considered the developed right renal vein. Supracardinal veins, like the other renal contributors, begin as bilaterally symmetric, having segmental connections with the posterior cardinal veins. As development continues, the posterior cardinal veins degenerate, losing the segmental nature of the connections that were established with the subcardinal and supracardinal network earlier. Ultimately, the supracardinal system contributes to the postrenal segment of the IVC. This segment fuses with the caudal posterior cardinal segment that, in turn, gives rise to the common iliac veins. Concomitantly, the supracardinal system on the left side loses connections with the developed left renal vein and the hemiazygos system. Persistent connections between the venous cardinal networks were observed in the current specimen.

Development of the ureters arises from bud outgrowths of the mesonephric duct. For each kidney, the ureteric bud penetrates the metanephric mesoderm (metanephrogenic blastema) that forms a cap into which the renal pelvis, calyxes, and collecting tubules develop through branching morphogenesis.^23^^-^ 25 The stalk of the ureteric bud elongates as the kidney undergoes its relative ascent, resulting in formation of the ureter by the ninth week of development. This simultaneous morphogenesis of the vascular and ureteric structures influences the medial rotation of the hilum for each developing kidney.^23^

## CONCLUSION

This case reports a unique anatomical variation showing non-rotation of a left kidney with a larger accessory renal artery and absence of normal renal pelvis. The embryological basis along with educational aspects described will provide depth of knowledge to medical graduates. Although this was a classic example of developmental kidney anomaly, there were no clinical manifestations which could be inferred from the case history of the donor. Alterations in relation of vascular structures and ureter at the hilum have great clinical significance when conducting surgical procedures like partial nephrectomy, nephron sparing surgery, and renal transplantation.
